# EORTC-1203-GITCG - the “INNOVATION”-trial: Effect of chemotherapy alone versus chemotherapy plus trastuzumab, versus chemotherapy plus trastuzumab plus pertuzumab, in the perioperative treatment of HER2 positive, gastric and gastroesophageal junction adenocarcinoma on pathologic response rate: a randomized phase II-intergroup trial of the EORTC-Gastrointestinal Tract Cancer Group, Korean Cancer Study Group and Dutch Upper GI-Cancer group

**DOI:** 10.1186/s12885-019-5675-4

**Published:** 2019-05-24

**Authors:** Anna Dorothea Wagner, Heike I. Grabsch, Murielle Mauer, Sandrine Marreaud, Carmela Caballero, Peter Thuss-Patience, Lothar Mueller, Annelie Elme, Markus Hermann Moehler, Uwe Martens, Yoon-Koo Kang, Sun Young Rha, Annemieke Cats, Masanori Tokunaga, Florian Lordick

**Affiliations:** 1 Department of Oncology, Lausanne University Hospital and University of Lausanne, Bugnon 46, 1011 Lausanne, Switzerland; 2 Department of Pathology and GROW School for Oncology and Developmental Biology, Maastricht University Medical Center+, Maastricht, Netherlands; 3 Division of Pathology and Data Analytics, Leeds Institute of Medical Research at St James’s, University of Leeds, Leeds, UK; 4 EORTC Headquarters, Avenue E. Mounier 83, 1200 Bruxelles, Belgium; 5 Department of Hematology, Medical Oncology and Tumor Immunology, Augustenburger Platz 1, Charité Universitätsmedizin Berlin, 13353 Berlin, Germany; 6 OnkologieUnterEms, 26789 Leer, Germany; 7 North Estonian Regional Hospital Cancer Center, Hiiu 44, 11619 Tallinn, Estonia; 8 University Medical Center, Johannes Gutenberg University Mainz, Langenbeckstr. 1, 55131 Mainz, Germany; 9 Department of Internal Medicine III, SLK-Kliniken Heilbronn GmbH, Am Gesundbrunnen 20-26, 74078 Heilbronn, Germany; 10 Department of Oncology, University of Ulsan College of Medicine, Asan Medical Center, Seoul, Republic of Korea; 11 Division of Medical Oncology, Department of Internal Medicine, Yonsei Cancer Center, Yonsei University College of Medicine, Seoul, Korea; 12 Department of Gastrointestinal Oncology, Netherlands Cancer Institute, Plesmalaaan 121, 1066 Amsterdam, CX Netherlands; 13 Division of Gastric Surgery, National Cancer Center Hospital East, 6-5-1, Kashiwanoha, Kashiwa, 277-8577 Japan; 14 University Cancer Center, University Medicine Leipzig, Liebigstr. 20, 04103 Leipzig, Germany

**Keywords:** Gastric cancer, Gastro-esophageal junction cancer, Perioperative chemotherapy, Trastuzumab, Pertuzumab, HER2

## Abstract

**Background:**

10–20% of patients with gastric cancer (GC) have HER2+ tumors. Addition of trastuzumab (T) to cisplatin/fluoropyrimidine-based chemotherapy (CT) improved survival in metastatic, HER2+ GC. When pertuzumab (P) was added to neoadjuvant T and CT, a significant increase in histopathological complete response rate was observed in HER2+ breast cancer. This study aims to investigate the added benefit of using both HER2 targeting drugs (T alone or the combination of T + P), in combination with perioperative CT for localized HER2+ GC.

**Methods:**

This is a prospective, randomized, open-label, phase II trial. HER2 status from patients with resectable GC (UICC TNM7 tumor stage Ib-III) will be centrally determined. Two hundred and-fifteen patients from 52 sites in 14 countries will be centrally randomized (1:2:2 ratio) to one of the following treatment arms:

1. ***Standard:*** CT alone. CT regimens will be FLOT (5-FU, leucovorin, oxaliplatin, taxotere) CapOx (capecitabine, oxaliplatin) or FOLFOX (5-FU, leucovorin, oxaliplatin) according to investigator’s choice in Europe, and cisplatin/capecitabine in Asia.
2. ***Experimental arm 1****:* CT as in control group, plus T (8 mg/kg loading dose, followed by 6 mg/kg every 3 weeks) at day 1, independent of CT chosen for 3 cycles of 3 weeks before and after surgery.
3. ***Experimental arm 2:*** CT plus T as in experimental arm 1, plus P (840 mg every 3 weeks) on day 1.

Adjuvant treatment with T or T + P will continue for 17 cycles in total. Stratification factors are: histology (intestinal/non-intestinal); region (Asia vs Europe); location (GEJ vs non-GEJ); HER2 immunohistochemistry score (IHC 3+ vs IHC 2+/FISH+) and chemotherapy regimen. Primary objective is to detect an increase in the major pathological response rate from 25 to 45% either with CT plus T alone, or with CT plus the combination of T and P.

**Discussion:**

Depending on the results of the INNOVATION trial, the addition of HER2 targeted treatment with either T or T and P to CT may inform future study designs or become a standard in the perioperative management HER2+ GC.

**Trial registration:**

This article reports a health care intervention on human participants and was registered on July 10, 2014 under ClinicalTrials.gov identifier: NCT02205047; EudraCT: 2014–000722-38.

## Background

Gastric cancer (GC) is the 5th most common malignancy, and third leading cause of cancer-related mortality worldwide [1]. Since the publication of the “MAGIC” trial [2], D2 resection in a high-volume surgical center and perioperative chemotherapy with epirubicin, cisplatin and 5-FU (ECF) became standard of care in Europe [3] for medically fit patients with resectable, T1N + M0 to T4N + M0 gastric cancer. In contrast, in Asia adjuvant chemotherapy with S-1 [4], or capecitabine and oxaliplatin [5] are standard of care for potentially curative resectable GC. The results of the “MAGIC” trial have recently been challenged by the presentation of the results from the AIO-FLOT-4-trial [6]. This randomized phase III study compared the perioperative chemotherapy regimens FLOT (5-FU, leucovorin, oxaliplatin and docetaxel) with ECF (epirubicine, cisplatin and 5-FU), and demonstrated a significant improvement in overall survival (median survival: 35 vs.50 months; projected 5-year-survival: 36 versus 45%) for patients treated with FLOT. Nevertheless, more than 40% of patients treated with perioperative FLOT have either a minimal or no pathological response to this treatment (TRG 3) (according to Becker et al. [7] or cannot proceed to surgery, and further improvement of therapy for GC patients is urgently required.

Between 10 and 20% of patients with GC are HER2 positive (HER2+) [8, 9]. In patients with advanced, HER2+ GC or gastro-esophageal junction (GEJ) adenocarcinoma, the addition of trastuzumab (T) to standard cisplatin/fluoropyrimidine chemotherapy improved overall survival significantly [10]. In HER2+ breast cancer, the addition of T to standard neoadjuvant chemotherapy significantly improved both, pathological complete response rates and event-free survival (defined as: time from randomization to disease recurrence or progression): 71% versus 56% at 3 years; Hazard Ratio (HR) = 0.59, *p* = 0.013) [11]. A further significant improvement of pathological complete response rates after neoadjuvant treatment of HER2 positive breast cancer has been observed recently, when the dimerization inhibitor pertuzumab was added to trastuzumab and chemotherapy (pathological complete response rates 29% versus 45%) [12]. In advanced, HER2+ breast cancer, the addition of pertuzumab to trastuzumab and docetaxel increased progression-free survival from 12.4 to 18.5 months (HR 0.62; 95% CI 0.51–0.75; *p* < 0.001) [13], and overall survival from 40.8 (95% CI 35.8–48.3) to 56.5 (95% CI 49.3 – not reached) months [14]. In contrast, in advanced HER2+ GC, the addition of pertuzumab to capecitabine/cisplatin/trastuzumab improved survival by 3.3 months, which did not reach statistical significance (*p* = 0.585) [15]. With respect to targeted, perioperative treatment of HER2+ GC and GEJ cancers, data from randomized clinical trials are lacking.

## Methods

### Study design and aim

EORTC 1203 -“INNOVATION” (https://www.eortc.org/research_field/clinical-detail/1203/) is a prospective, multicenter, international, randomized phase II trial which aims to evaluate the effect of targeted treatment using either trastuzumab, or the combination of trastuzumab plus pertuzumab in combination with perioperative chemotherapy on the pathologic response rate in patients with localized (stage IB-III according to TNM, 7TH edition) GC or GEJ adenocarcinoma. The trial was designed by the EORTC, which is the legal sponsor. It is conducted by EORTC as leading group, in cooperation with the Korean Cancer Study Group (KCSG) and the Dutch Upper Gastrointestinal Cancer Group (DUCG). Surgical quality assessment is performed in collaboration with the Japanese Clinical Oncology Group (JCOG).

### Study flow

**Fig. 1:**
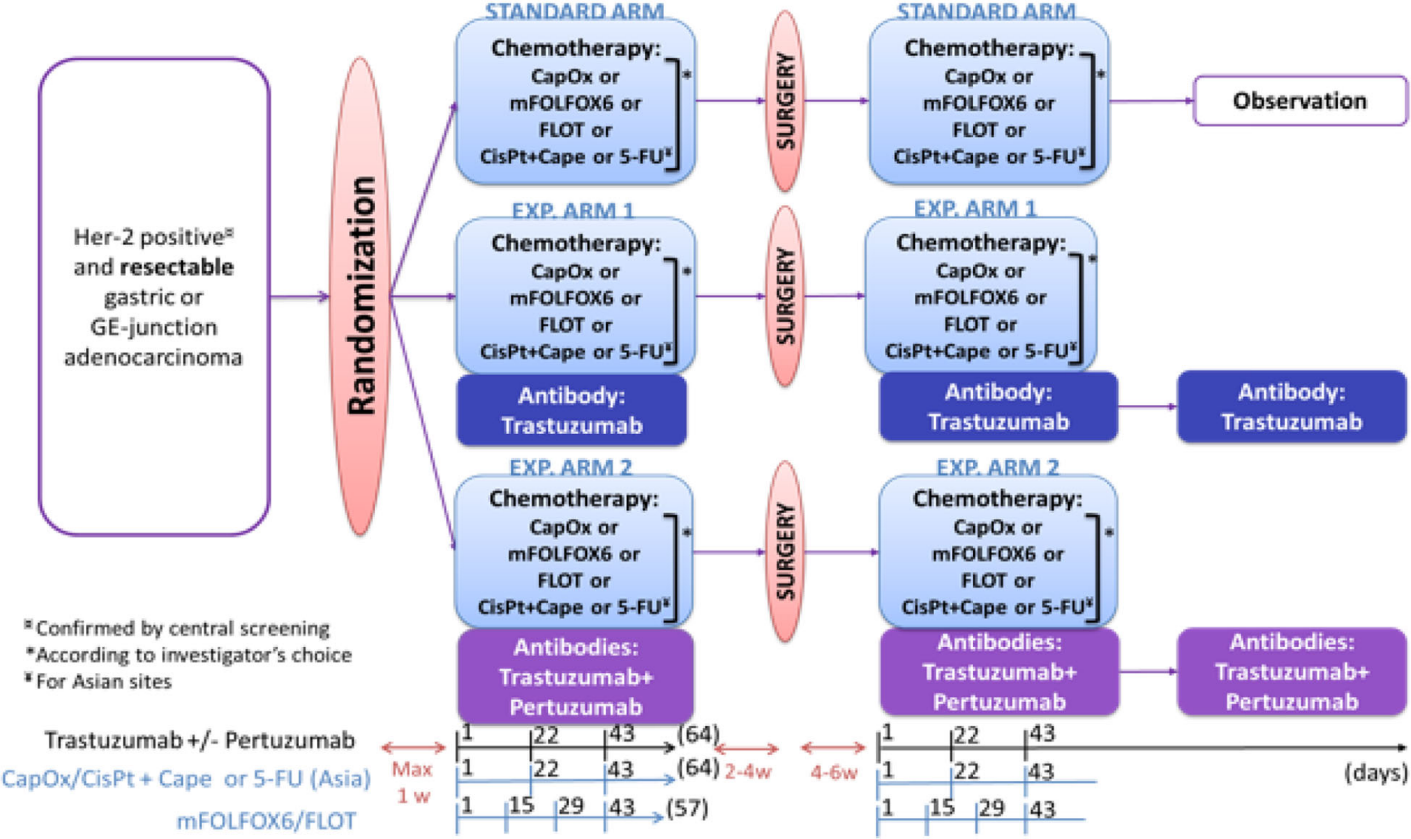
INNOVATION Study (Version 4.1) flow chart. CapOx: Capecitabine, Oxaliplatin; mFOLFOX 6: Modified FOLFOX 6; FLOT: 5-fluorouracil (FU), Leucovorin, Oxaliplatin and Taxotere; CisPT: Cisplatin; Cape: Capecitabine; Her-2: Human epidermal growth factor receptor type 2; GE-junction: Gastroesophageal junction; w: weeks

After signing the informed consent and registration of potentially eligible patients, formalin fixed paraffin embedded material from the diagnostic biopsy is sent to a central laboratory for determination of the HER2 status. Only patients with a centrally determined, positive HER2 status, who fulfill the eligibility criteria mentioned below, are randomized centrally by the EORTC randomization system in the following three treatment arms (Fig. 1):

**Interventions:** In the initial version of the protocol (version1.0 of August 29, 2014), based on the evidence available at that time, cisplatin and 5-FU/capecitabine [16] were selected as chemotherapy backbone. After publication of the results of the FLOT-4 trial [17], the protocol was amended (version 4.1 of 11/12/2017) to allow - in Europe – either FLOT, FOLFOX or CapOx as chemotherapy backbone, according to what the investigator considers as in the best interest of the patient. By contrast, in absence of data for FLOT in Asian patients, and after discussion with the Asian investigators, the chemotherapy backbone of cisplatin and 5-FU/capecitabine in Asian patients remains unchanged. A detailed list of all protocol changes is provided in Appendix 2.

#### Standard arm - chemotherapy alone

- ***Before amendment 4.1*** (*and continued in Asia after this amendment*): Cisplatin (80 mg/m^2^ every 3 weeks) and capecitabine (1000 mg/m^2^ twice daily every 2 out of 3 weeks), or 5-FU (800 mg/m^2^/day for 5 days every 3 weeks) for 3 cycles before and after surgery.
- ***After amendment 4.1*** in Europe**: FLOT** is administered in cycles of 2 weeks for 4 cycles (= 8 weeks) on day 1, 15, 29 and 43 pre- and postoperatively, with Docetaxel 50 mg/m^2^, followed by Oxaliplatin 85 mg/m^2^ diluted with 250 to 500 ml of 5% glucose solution as a 2 h infusion, leucovorin 200 mg/m^2^ over 2 h and 5-FU 2600 mg/m^2^ as a 24 h-infusion. Alternatively, either **CapOx** is given for 3 cycles of 3 weeks (=9 weeks) on day 1, 22 and 43 pre- and postoperatively, with Oxaliplatin 130 mg/m^2^ on day 1, and followed by capecitabine given orally at a dose of 1000 mg/m^2^ twice daily from the evening of day 1 to the morning of day 15 every 3 weeks or **mFOLFOX6** is given for 4 cycles of 2 weeks (=8 weeks) on day 1, 15, 29 and 43 pre- and postoperatively, with oxaliplatin at a dose of 85 mg/m^2^, followed by leucovorin 400 mg/m^2^ iv over 2 h on day 1, and 5-FU 400 mg/m^2^ iv bolus on day 1, then 1200 mg/m^2^/d × 2 days over 46–48 h continuous infusion every 2 weeks. Chemotherapy should be restarted 4 to 6 weeks after surgery, if the patient has sufficiently recovered, in all trial arms

#### Experimental arm 1

Chemotherapy as in standard arm, plus trastuzumab (8 mg/kg loading dose, followed by 6 mg/kg every 3 weeks) on day 1, 22 and 43, independent of the chemotherapy regimen chosen, for 3 cycles of 3 weeks before and after surgery.

#### Experimental arm 2

Chemotherapy plus trastuzumab as in experimental arm 1, plus pertuzumab (840 mg every 3 weeks) at day 1, 22 and 43, independent of the chemotherapy regimen chosen.

**Surgery** is scheduled within 2–4 weeks after the completion of cycle 3 in a 3-week-cycle, and after completion of cycle 4 in a 2-week-cycle if the white blood count has normalized again and the patient is clinically deemed fit to undergo major surgery. Surgery is performed according to the Japanese Gastric Cancer Treatment Guidelines 2014 (version 3) [18]. The extent of the surgical resection depends primarily on the location of the tumor and is either an extended total, partial or subtotal gastrectomy, or – for tumors of the GEJ – esophagogastrectomy and reconstruction via gastric tube or extended total gastrectomy according to the decision of the surgeon. Surgical CRFs, mandatory intra-operative photo documentation, the operative report, the pathology report, central pathology review and assessment of surgical complications according to Dindo [19] will be used for surgical quality assessment.

**Maintenance treatment** is performed with trastuzumab alone or trastuzumab plus pertuzumab in experimental arms 1 and 2 starting after the end of adjuvant chemotherapy, and continuing for a total of 17 cycles from the start of neoadjuvant treatment.

### Main inclusion criteria for REGISTRATION

Histologically proven, gastric or GEJ adenocarcinoma (Siewert I-III), absence of distant metastases on CT scan of thorax and abdomen, age > 18 years, WHO performance status 0–1 in a patient judged medically fit for gastrectomy/esophagogastrectomy as decided by the investigator.

### Main inclusion criteria for RANDOMIZATION

HER2 overexpression, as determined by central testing using immunohistochemistry (IHC 3+) or the combination of IHC 2+ and HER2 FISH (Fluorescence In Situ-hybridization) positive. Amenable to gastrectomy/esophagogastrectomy with curative intent as confirmed by a multidisciplinary team discussion. UICC (7th edition) tumor stage Ib to III, as defined by CT scan and/or MRI. Endosonography (EUS) is recommended, but not mandatory. Absence of contraindications for any of the study treatments. No prior chemotherpy or antibody treatment. Adequate organ function. Written informed consent according to ICH/GCP, and national/local regulations.

**Primary endpoint:** major pathological response rate (< 10% vital residual tumor cells) according to Becker et al. [20].

**Secondary endpoints:** are R0 resection rate, pathological complete response rate, locoregional failure, distant failure, progression-free survival according to RECIST v1.1, recurrence-free-survival from surgery, overall survival and adverse events (according to CTCAE v 4.0).

### Schedule of investigations

**Before registration:** histologic diagnosis of gastric or GE-junction adenocarcinoma, written informed consent for screening.

**During screening:** Central HER2 assessment, CT scan of thorax and abdomen (needed within 35 days of treatment start), hepatic MRI in case of suspected liver lesions, brain MRI if clinically indicated echocardiography, ECG, laboratory tests including hematology and biochemistry, assessment of adverse events, multidisciplinary team discussion of surgical resectability and general operability. Echo-endoscopy and laparoscopy are not mandatory and performed at the investigator’s discretion.

**After randomization/before each (neo) adjuvant chemotherapy cycle:** Clinical and laboratory tests, including hematology and biochemistry, assessment of adverse events.

**Before surgery:** Multidisciplinary team discussion of surgical resectability and general operability, clinical and laboratory tests, including hematology and biochemistry, assessment of adverse events.

**During maintenance treatment:** Clinical and laboratory tests, including cardiac evaluation by ECG and echocardiography every three months.

**During adjuvant/maintenance treatment and follow-up:** CT-scan of thorax and abdomen every 6 months in year 1 and 2, yearly thereafter.

**Sample size calculation and analysis populations:** Based on the results in breast cancer, where the addition of trastuzumab to neoadjuvant treatment with docetaxel increased the total pathologic complete response in the primary tumor and axillary nodes (tpCR) from 19 to 38% [11], and where the addition of pertuzumab to the combination of trastuzumab and chemotherapy further increased pathologic tpCR from 31 to 45% [12], the primary objective of the “INNOVATION” trial is to detect an increase in the major pathological response rate of the primary tumor from 25 to 45% either with chemotherapy plus trastuzumab alone, or with chemotherapy plus the combination of trastuzumab and pertuzumab. A comparative design with an increased one-sided type I error of 10% will be applied to test each experimental arm versus the control arm. To have 80% power to detect an increase from 25 to 45% in major pathological response rate between the control arm and each experimental arm using a Z-test for the comparison of two sample proportions with a one-sided type I error of 10%, 38 patients are required in the control arm and 76 patients in each experimental arm. A stepwise testing procedure will be used in order to avoid inflation of the one-sided type I error due to multiple testing. Therefore, the hypothesis of an increase in major pathological response rate with both trastuzumab and pertuzumab will first be tested using a one-sided type I error of 10%. If significant, the hypothesis of an increase in major pathological response rate with trastuzumab alone will be tested. As such, the overall one-sided type I error will be kept below 10%. Analysis populations are the following:

- Per protocol population: All patients who are eligible and have started their allocated treatment (at least one dose of the study drug(s) planned as pre-operative treatment).
- Safety population: All patients who have started their allocated treatment (at least one dose of the study drug(s) planned as pre-operative treatment).
- Resected population: All eligible patients who have started the allocated pre-operative treatment, were operated and achieved a R0 or R1 resection.

The primary analysis of the major pathological response rate will be performed in the per protocol population with a non-missing assessment of pathological response. This includes all patients in the per protocol population who have had a resection and for whom the tumor regression according to Becker et al. has been assessed by the central pathology laboratory or who did not proceed to surgery or did not have a resection for any reason and therefore will be considered as not having a major pathological response in the analysis. Thus, missing data for the primary endpoint will only occur if a patient has had a resection and the central pathology review of the resection specimen is not possible for technical reasons e.g. if the resection specimen is not provided at all by the local pathologist or are not provided in sufficient quality or quantity. These patients will be excluded from the analysis. All reasonable efforts will be made to minimize this risk, e.g. the local pathologists will receive detailed information (written pathologist guidelines, web-based training) about the material and procedure necessary for central pathology review and will be asked to sign that he agrees to provide the material required according to protocol during the evaluation of the feasibility of the study in a center. Assessment of pathologic response will be performed independently by two senior pathologists specialized in gastrointestinal cancers, who will be blinded to treatment allocation. Inter-observer variability will be adjucidated by a third expert pathologist. In contrast, caregivers and patients will not be blinded to the allocated treatment. Randomization (both generation of the allocation sequence and assignment of participants to interventions) will be performed centrally by the EORTC computerized randomization system. Randomization between the three arms will continue until the required number of patients for the primary analysis is reached (38 patients in the control arm and 76 patients in each experimental arm). Using a safety margin of 10% in each arm, a maximum of 215 patients will have to be randomized in total. After analysis of the primary endpoint the advice of the EORTC Independent Data Monitoring Committee (IDMC) (https://www.eortc.org/governance/committees/) will be sought regarding the possible conduct of a phase III trial. Follow up will continue until death or for a minimum of 6 years after end of treatment.

### Ethical considerations

The study protocol has been approved by the competent ethics committee as requested by the applicable national legislation. It is conducted in agreement with either the Declaration of Helsinki (available on the World Medical Association web site [21] and/or the laws and regulations of the country, whichever provides the greatest protection of the patient as well as according to the ICH Harmonized Tripartite Guideline on Good Clinical Practice (ICH-GCP), available online at [22].

**Written informed consent** according to the above mentioned regulations is obtained prior to any study-related procedure by the investigator in each participating center.

## Discussion

The development of HER2 targeting treatments for breast cancer is one of the greatest achievements in modern oncology, and permitted to improve median survival for patients with metastatic, HER2+ breast cancer from about 12 months to more than 50 months in a recently published trial [14].

Subsequently, HER2 targeting treatments were also developed in gastric cancer. Trastuzumab was the first targeted agent, which – when added to palliative chemotherapy – demonstrated a significant survival benefit in patients with metastatic GC [10], without the side effects of chemotherapy or other new safety signals. As such, it was a milestone in drug development. Whereas pertuzumab significantly improved survival in patients with metastatic breast cancer, this was not the case in patients with metastatic GC as reported in the JACOB-trial recently. Despite a survival benefit of 3.3 months, this was not statistically significant according to the hypothesis specified in the protocol. However, the question whether the integration of HER2 targeting drugs in perioperative chemotherapy may further improve treatment outcomes in patients with localized or locally advanced resectable GC remains one of the major open questions in the current management of GC patients. While trastuzumab is certainly the most promising drug to study in this indication, pertuzumab might still be able to increase the benefit of trastuzumab when given at an earlier time point in the evolution of this disease and for a longer time. The “INNOVATION”-trial has the advantage to assess the relative benefit of both trastuzumab and pertuzumab individually in the perioperative treatment of GC patients. In addition, as not all patients are eligible for treatment with FLOT, and given the currently limited experience of FLOT outside of Germany at present, “INNOVATION” will allow a preliminary estimation of the relative benefit of these antibodies with different chemotherapy backbones (CF and FOLFOX or CapOx).

In addition to systemic treatment, it is well established that the quality of surgery has a major impact on the patients’ outcome. Surgical quality assurance is therefore another key feature of this study protocol. An integrated approach [23, 24] has been adapted for this study through the central review by independent surgeons and pathologists from JCOG and EORTC. Data from surgical and pathology case report forms, pathology review, intraoperative photographs and macroscopic pictures of the resected specimen will be reviewed to assess completeness of surgical treatment. Thirty and 90 day surgical complications, graded by the Clavien-Dindo Classification [19], will also be reviewed.

Finally, assessment of histopathological tumor regression may be challenging as different tumor regression systems are used by local pathologists. To minimize the risk of bias, the primary endpoint, major pathological response, will be assessed centrally, by two expert gastrointestinal pathologists, according to a previously published, validated protocol [20], with discrepancies being resolved by a third expert gastrointestinal pathologist. As major pathological response rate remains a surrogate endpoint, the final decision to proceed with further development of trastuzumab and pertuzumab, either alone or in combination, in this indication will also consider other endpoints, such as safety, progression- free survival and overall survival.

Furthermore, there is evidence to suggest that the biology of gastric cancer biology differs significantly between Asian and Caucasian patients [25], and a large variability in the results of surgery [2, 4, 26] and several systemic treatments [27, 28] between different geographical regions has been observed since years. Thus, it is unclear if treatment benefits observed in one region of the world will have the same magnitude in other regions.

“INNOVATION” is a unique example of an academic, international, multidisciplinary collaboration of four leading academic groups in upper GI-cancer: EORTC, DUCG and KCSG, with JCOG being responsible for the surgical quality assurance, under the leadership of EORTC. Due to its design and recruitment in different European countries, as well as Korea and Singapore, “INNOVATION” will permit to estimate the relative benefit of perioperative HER2 targeted treatment with trastuzumab and pertuzumab individually in different geographical regions, and with different chemotherapy backbones and provide a rational basis for the design of future confirmatory studies in patients with resectable GC.

## Appendix 1

**Item 2b: All items from the WHO Trial Registration Data Set** (if not described in the protocol text).

**Primary registry and trial identifying number, including date of registration and secondary identifying numbers:** see abstract.

**Primary sponsor:** European Organization for Research and Treatment of Cancer (EORTC), Avenue E. Mounierlaan 83/11, 1200 Bruxelles, Belgique. Email:eortc@eortc.be, https://www.eortc.org.

**Secondary sponsor: see declarations.** The trial is supported by an educational grant by F. Hoffmann La Roche to EORTC.

**Contact for public queries:** 1203@eortc.be

**Contact for scientific queries**: 1203@eortc.be

**Public title:** EORTC-1203-GITCG: INNOVATION-trial.

**Scientific title:** Integration of trastuzumab, with or without pertuzumab, into perioperative chemotherapy of HER-2 posiTIve stomach cancer: the INNOVATION-TRIAL.

**Countries of recruitment (as of 19.09.2018):** South Korea, Germany, Estonia, United Kingdom, Italy, Spain, France, Israel, Switzerland, Norway, Portugal, Belgium.

**Health condition or problem studied:** HER-2 positive, gastric and gastroesophageal junction cancer.

**Interventions:** see methods.

**Key inclusion and exclusion criteria:** see methods.

**Study type:** Interventional, Allocation: randomized. Masking: single blind (outcomes assessor). Primary purpose: treatment, phase II.

**Date of first enrolment: 01.12.2015.**

**Target sample size: n=215**

**Recruitment status:** 69 patients as of 19.09.2018.

**Primary outcomes:** see methods.

**Key secondary outcomes: see methods**

## Appendix 2

### Summary of protocol changes from protocol v3.0 to 4.0

- Change of chemotherapy backbone: in Europe, the regimen will be selected between FLOT, CapOX por mFOLFOX6; in Asia, cisplatin +capecitabine or 5-FU.

This scientific amendment was necessary after the presentation of the results of the FLOT-4-trial at ASCO 2017, which demonstrated a significantly better progression-free and overall survival for perioperative treatment with FLOT, as compared to ECF. Both, dose modification and supportive care recommendations for treatment with FLOT have been inspired by those used in the FLOT-4 study, which were generously provided by the author. Cisplatin is being kept as an option as it is still standard of care in Korea, and efficacy benefit of other regimen yet have to be demonstrated in these patients (section 4 trial design, page 28; therapeutic drug regimen, section 5; newly added Appendix L).

- A safety run has been introduced in the protocol, given the change of background chemotherapies (section 4.1).

- A definition of ‘high dose corticosteroids’ was inserted (section 3.2).

- Clarification was added, that DPD testing should be done according to local guidelines (section 3.2; section 5.4.5.1.1).

- Dose reduction upon grade 2 or 3 nausea or vomiting has been adapted (appendix L for Cisplatin-treated patients).

- Recommendations regarding the procedures in case of tubular damage and low Mg/Ca were added in Table 9 (Appendix L).

- Measures to be taken after assessment of ototoxicity and sensory neural damage were clarified (Appendix L, section ‘Other precautions’).

Furthermore:

- The numbers of treatment cycles and timelines for follow-up have been clarified and made consistent between the protocol and PISIC.

- For enrolled patients, the side effects and toxicities have been updated in the protocol and PISIC based on the new versions of the IBs of Pertuzumab.

and Trastuzumab.

- A PISIC specific to the new chemotherapy backbone has been created, while the Cisplatin-only PISIC remains valid for Asian patients, as well as already registered patients.

- The RECIST criteria have been added in the protocol.

- Additional clarifications, update of contact information and administrative corrections have been implemented.

**Summary of changes from protocol v4.0 to 4.1:**

Clarifications included in the protocol about:

- Time Window for tests during the follow up period.

- Treatment administration in case of delay for initiating the adjuvant treatment.

## Abbreviations

Cape: Capecitabine
CapOx: Capecitabine, Oxaliplatin
CisPT: Cisplatin
CT: Chemotherapy
CT: Computed tomography
CTCAE: Common Terminology Criteria for Adverse Even
DUCG: Dutch Upper Gastrointestinal Cancer Group
ECF: Epirubicine, Cisplatine and 5-FU
EORTC: European Organization for the Research and Treatment of Cancer
EUS: Endoscopic ultrasound
FISH: Fluorescence in situ hybridization
FLOT: 5-fluorouracil (FU), Leucovorin, Oxaliplatin and Taxotere
FOLFOX: 5-FU, Leucovorin, Oxaliplatin
GC: Gastric cancer
GCP: Good clinical practice
GEJ: Gastroesophageal junction
HER2: Human epidermal growth factor receptor type 2
ICH: International Conference on Harmonization of Technical Requirements for Registration of Pharmaceuticals for Human Use
JCOG: Japanese Clinical Oncology Group
KCSG: Korean Cancer Study Group
mFOLFOX 6: Modified FOLFOX 6
MRI: Magnetic resonance imaging
P: Pertuzumab
RECIST: Response Evaluation Criteria In Solid Tumors
T: Trastuzumab
UICC: International Union Against Cancer

## Authors’ information

ADW had the idea for this trial, has been coordinating the protocol development, is the coordinating investigator and responsible for the present paper. She is a senior lecturer and consultant at the University Hospital of Lausanne, where she is head of the upper gastrointestinal cancer unit in the department of oncology. In addition, she is chair of the EORTC GI GROUP gastric task force (together with MHM).
